# Oblique Bile Duct Predisposes to the Recurrence of Bile Duct Stones

**DOI:** 10.1371/journal.pone.0054601

**Published:** 2013-01-24

**Authors:** Pavel Strnad, Guido von Figura, Regina Gruss, Katja-Marlen Jareis, Adolf Stiehl, Hasan Kulaksiz

**Affiliations:** 1 Department of Internal Medicine I, University Medical Centre Ulm, Ulm, Germany; 2 Department of Internal Medicine III and IZKF, University Hospital Aachen, Aachen, Germany; 3 Department of Internal Medicine I, Division of Gastroenterology, University Medical Centre Heidelberg, Heidelberg, Germany; 4 Department of Internal Medicine II, Hospital Waldshut-Tiengen, Waldshut-Tiengen, Germany; University of Chicago, United States of America

## Abstract

**Background and Study Aims:**

Bile stones represent a highly prevalent condition and abnormalities of the biliary tree predispose to stone recurrence due to development of biliary stasis. In our study, we assessed the importance of an altered bile duct course for stone formation.

**Patients and Methods:**

1,307 patients with choledocholithiasis in the absence of any associated hepatobiliary disease who underwent endoscopic retrograde cholangiopancreatography (ERCP) between 2002 and 2009 were analysed. The angle enclosed between the horizontal portion of the common bile duct (CBD) and the horizontal plane was measured (angle α). Oblique common bile duct (OCBD) was defined as a CBD with angle α<45°.

**Results:**

103 patients (7.9%) were found to harbour OCBD and these were compared to 104 randomly selected control subjects. Compared to controls, OCBD patients were (i) significantly older (72±13 vs. 67±13, p<0.00001); (ii) more frequently underwent a cholecystectomy (p = 0.02) and biliary surgery (p = 0.003) prior to the diagnosis and (iii) more often developed chronic pancreatitis (p = 0.04) as well as biliary fistulae (p = 0.03). Prior to and after ERCP, OCBD subjects displayed significantly elevated cholestatic parameters and angle α negatively correlated with common bile duct diameter (r = -0.29, p = 0.003). OCBD subjects more often required multiple back-to-back ERCP sessions to remove bile stones (p = 0.005) as well as more ERCPs later on due to recurrent stone formation (p<0.05).

**Conclusion:**

OCBD defines a novel variant of the biliary tree, which is associated with chronic cholestasis, hampers an efficient stone removal and predisposes to recurrence of bile duct stones.

## Introduction

Gallstones represent a highly prevalent condition in the Western world, affecting up to 20% of the population [1]–[3]. 10–15% of the symptomatic patients have concomitant common-bile duct (CBD) stones [1], [2], which can remain asymptomatic; however, they carry an increased risk of serious complications such as biliary obstruction, acute cholangitis and pancreatitis [1], [2]. In the Western world, the majority of CBD stones originate from the gallbladder and consist primarily of cholesterol [2]. However, primary CBD stones, which are more common in East Asia, are brown-pigmented and are formed in the bile duct as a consequence of bile stasis and infections [4], [5].

Endoscopic retrograde cholangiopancreatography (ERCP) is the treatment of choice for symptomatic CBD stones [1], [3]. However, a recurrence of stones after ERCP is reported in 4–24% of patients [7]–[9], and patients with recurring CBD stones are at increased risk for a subsequent stone re-formation [4]. Given the potential serious complications of CBD stones, ranging from acute cholangitis/pancreatitis to asymptomatic development of secondary biliary cirrhosis [4], [6], an identification and careful follow-up of patients with recurring stones is necessary [7], [8]. Congenital and acquired risk factors are important in the pathogenesis of primary CBD stones [2], [4]. In addition, disorders such as ampullary stenosis, periampullary diverticula, cholangiocarcinoma, haemolytic anaemia and also various congenital abnormalities of the biliary tree, including choledochal cyst, choledochocele or Caroli disease, can lead to bile stasis and colonisation of the bile with enteric organism, thereby contributing to the formation of primary bile duct stones [4], [10].

In the present study, we describe a new entity of the biliary tree, which was termed the oblique common bile duct (OCBD) and is characterised by a horizontal common bile duct course. OCBD was found to be associated with chronic cholestasis, hampers an efficient stone removal and predisposes to recurrence of bile duct stones.

## Materials and Methods

### Ethics Statement

The study was approved by the Human Subject Committee of Ulm University, which allowed the retrospective analysis of the data and permitted us to approach the selected patients to obtain further data. An informed written consent was obtained from all subjects who were part of the control and study groups and from whom additional clinical data have been obtained.

### Patients and Study Design

In this retrospective study, we analysed all patients who underwent endoscopic retrograde cholangiopancreatography (ERCP) in the Department of Internal Medicine I at University Medical Centre Ulm between January 2002 and December 2009 (2,459 patients). All subjects with choledocholithiasis with the absence of any associated hepatobiliary disease such as primary sclerosing cholangitis, bile duct stenosis, hepatobiliary malignancies or diverticula were included (1,307 patients, Table S1). ERCP with endoscopic sphincterotomy was performed under intravenous sedation. Antibiotics were given when appropriate. A complete endoscopic sphincterotomy was performed after deep cannulation of the CBD with a long-nose papillotome (Olympus, Hamburg, Germany) and pre-cut sphincterotomy was done when deep CBD cannulation failed. Retrieval Dormia baskets and/or balloons were used for stone extraction. Patients with large stones (>1–1.5 cm in diameter) underwent endoscopic mechanical lithotripsy (Wilson-Cook, Bloomington, IN) or extracorporeal shock wave lithotripsy (Lithostar Plus; Siemens, Erlangen, Germany). Balloon-occlusion cholangiogram was performed at the end of stone extraction to exclude the presence of residual stones in the bile ducts.

**Figure 1:**
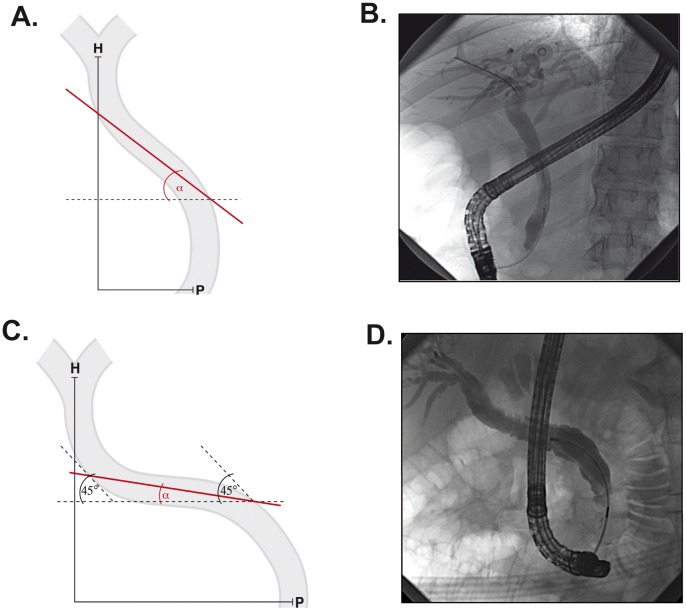
Characterisation of the oblique common bile duct syndrome. Schematics (A,C) and radiographs (B,D) depict the morphology of “normal” (A,B) and “oblique” bile ducts (C,D). Angle α was defined as the angle enclosed between the horizontal portion of the bile duct and the horizontal plane. H, porta hepatis; P, papilla Vateri.

CBD morphology was analysed in all 1,307 study patients. In most of them, CBD was running straight upward towards the porta hepatis (Fig. 1), while in others an altered CBD course was observed. In the latter subjects, at least a portion of CBD often enclosed an angle of less than 45° with the horizontal plane (Fig. 1). Such a CBD was termed “oblique” and the minimal angle between the horizontal plane and the CBD was determined (Fig. 1). Of note, some of the CBDs harboured a downward-facing part and consequently enclosed a negative angle with the horizontal plane (Fig. S1). To accurately determine the angle, a standardised position was used in all subjects. In this position, the patients were lying face-down and the picture was taken in an anterior-posterior axis.

To determine the significance of “oblique” CBDs, a variety of clinical parameters was compared between the patients with “oblique” CBDs and a control group that was randomly selected from the remaining study subjects. The charts from the initial hospital stay and subsequent treatments were reviewed and recorded. Follow-up data were collected by personal interview, mail or telephone call to the patients. All patients were asked about the recurrence of biliopancreatic symptoms and further interventional procedures. Blood parameters were requested from the primary care physicians. In deceased patients, the clinical history was collected from the relatives and/or the primary care physicians.

### CBD Parameters and Statistical Analyses

Maximum CBD diameter and other parameters of the “oblique” CBDs were measured manually on the ERCP radiograph and corrected according to the scope diameter. In patients who underwent more ERCPs during the course of the study, the first ERCP was used to determine CBD parameters and to find out whether patients harboured an “oblique” CBD. All data were expressed as means±SD or as median values and quartiles. Statistical analysis was performed with a two-tailed Chi-square, Fisher’s exact, Student’s *t* or Mann-Whitney U tests. A *P* value of less than 0.05 was considered statistically significant. To determine a correlation between two variables, the Pearson Product Moment Correlation test for ungrouped data was employed (SPSS software, version 17.0, SPSS, Chicago, IL, USA).

## Results

Between 2001 and 2009, a total of 2,459 patients underwent an ERCP at the University Medical Centre Ulm. Among them, 1,307 met the study inclusion criteria and 103 (7.9% of the study subjects) presented with an “oblique” CBD (OCBD; Table S1). Of note, OCBD patients were significantly older (p<0.00001), while no difference was seen in the gender distribution (Table S1).

To determine the events leading to OCBD, past medical history was analysed in OCBD patients and a control group consisting of 104 subjects randomly selected from the remaining study population (Table 1). Prior to the diagnosis, OCBD subjects more frequently underwent a cholecystectomy (p = 0.02) and were more likely to require an open (vs. laparoscopic) cholecystectomy (p = 0.005). Prior CBD surgery was also more commonly seen in OCBD subjects (p = 0.003), suggesting that in some cases OCBDs may develop secondary to a biliary affection (Table 1). This hypothesis was strengthened by the observations during ERCP, where OCBD subjects more commonly suffered biliary fistulae and chronic pancreatitis (Table 2). To test whether OCBD constitutes a progressive condition, we compared angle α in 14 subjects who underwent more than one ERCP that were at least one year apart. However, no difference in angle α was noted despite a median time of 3.8 years between the procedures (Table S2).

**Table 1:** Prior medical history.

	“Oblique” CBD	Controls
Prior cholecystectomy (n), yes/no	56/40^1^	42/58^1^
Type of cholecystectomy (n), laparoscopic/open	26/41^2^	49/30^2^
**Comorbidities:**		
*1.Metabolic syndrome*		
Obesity-BMI>30 kg/m2 (n), yes/no	24/79	30/74
High blood pressure (n), yes/no	55/48	56/48
Diabetes mellitus	24/79	14/90
Hyperlipoproteinemia	17/86	12/92
*2. Cardiovascular system*		
Hematologic disorders (n), yes/no	7/96	2/102
Heart disease (n), yes/no	37/66	32/72
Vascular disorders (thrombembolic, aneurysms etc.) (n), yes/no	13/90^3^	5/99^3^
*3. Digestive system*		
Gastroesophageal reflux disease (n), yes/no	5/98	11/93
Gastric disorders (ulcers, gastritis) (n), yes/no	17/86	19/85
Bowel disorders (n), yes/no	11/92	6/98
*4. Respiratory system*		
COPD, Asthma (n), yes/no	7/96	8/96
*5. Genitourinary impairments*		
Chronic kidney disease (n), yes/no	15/88	9/96
*6. Neurological/psychiatric disorders*		
Stroke (n), yes/no	6/97	2/102
Psychiatric disorders (n), yes/no	12/91	5/99
**Abdominal surgery**		
None (n), yes/no	75/28	87/17
Common bile duct surgery (n), yes/no	8/95^4^	0/104^4^
Colorectal surgery (n), yes/no	5/98	5/99
Hysterectomy (n), yes/no	5/98	4/100
Gastric surgery (n), yes/no	3/100	0/104
Nephrectomy (n), yes/no	1/102	1/103
Hepaticojejunostomy (n), yes/no	3/100	0/104

BMI: body mass index; CBD: common bile duct; COPD: chronic obstructive pulmonary disease.

1 p = 0.02;

2 p = 0.005;

3 p = 0.05;

4 p = 0.003;

**Table 2:** ERCP findings.

ERCP findings	“Oblique” CBD	Controls
Common bile duct stones (n),yes/no	103/0	104/0
Acute cholangitis (n), yes/no	42/61	31/73
Biliary pancreatitis (n), yes/no	21/82	23/81
Chronic cholangitis (n), yes/no	4/99	1/103
Biliary fistula (n), yes/no	5/98^1^	0/104^1^
Chronic pancreatitis (n), yes/no	7/96^2^	1/103^2^
Hepatopathy (n), yes/no	4/99	3/101
Papillary stenosis/−sclerosis (n),yes/no	4/99	0/104

1 p = 0.03;

2 p = 0.04.

To analyse how the presence of OCBD affects the development and resolution of biliary disease, we analysed the lab values before and after the ERCP procedure (Table 3). Surprisingly, shortly before and after ERCP, OCBD subjects presented with lower alanine aminotransferase levels; however, they had higher C-reactive protein levels, suggesting a higher susceptibility to development of acute cholangitis. The latter observation was further supported by ERCP findings (i.e., a trend towards a more common acute cholangitis in OCBD subjects, Table 2, p-value<0.1). However, >1 week before/after ERCP, OCBD subjects displayed significantly higher levels of cholestatic enzymes (bilirubin, γ-glutamyl transferase and alkaline phosphatase), indicating that OCBD may predispose to chronic cholestasis (Table 3). Of note, similar findings were obtained from a subgroup of patients who underwent cholecystectomy prior to the ERCP, confirming that the stronger chronic cholestasis is not due to a more common removal of the gallbladder in the OCBD subjects (Table S3).

**Table 3:** Lab values before and after endoscopic retrograde cholangiopancreatography.

	AP (U/l)		GGT (U/l)		Bili (µmol/l)		AST (U/l)		ALT (U/l)		CRP (mg/l)	
	OB	Co	OB	Co	OB	Co	OB	Co	OB	Co	OB	Co
**Before ERCP**												
>3 mo	99^1^	82^1^	29^2^	18^2^	11	10	18	15	16	16	3	7
1–12 wk	142^3^	96^3^	72^4^	28^4^	19^5^	8^5^	45^6^	20^6^	31	20	26	8
4–7d	163	134	188	269	13	20	34	57	36	77	15	25
1–2d	189	173	327	280	30	32	86	119	76^7^	185^7^	14	16
**ERCP**												
	201^8^	157^8^	272	364	54	34	80	92	98^9^	190^9^	91^10^	27^10^
**After ERCP**												
1d	195	152	277	375	37	35	76	77	99^11^	182^11^	59	56
2d	162^12^	100^12^	292	279	49^13^	19^13^	65	63	75	137	105	63
3d	168	135	219	266	22	21	32	34	46^14^	94^14^	35	60
4–7d	152	146	189	224	17	12	38	33	39^15^	61^15^	26	32
1–12 wk	99^16^	82^16^	83	60	9	9	22	24	21^17^	31^17^	11	20
>3 mo	91^18^	75^18^	42^19^	24^19^	10	8	25^20^	22^20^	17	20	14	6

Median values are shown. Calculation of statistical significance is based on a two-tailed Mann-U-Whitney test.

AP, alkaline phosphatase; GGT, gamma glutamyl transferase; Bili, bilirubin; AST, aspartate transaminase; ALT, alanine transaminase; AP, alkaline phosphatase; d, day; wk, week; mo, month; ERCP, endoscopic retrograde cholangiopancreatography; OB, “oblique” bile duct; Co, Controls.

1 p = 0.008;

2 p = 0.01;

3 p = 0.05;

4 p = 0.05;

5 p = 0.01;

6 p = 0.007;

7 p = 0.003;

8 p = 0.03;

9 p = 0.009;

10 p = 0.03;

11 p = 0.02;

12 p = 0.02;

13 p = 0.01;

14 p = 0.02;

15 p = 0.01;

16 p = 0.02;

17 p = 0.01;

18 p = 0.06;

19 p = 0.02;

20 p = 0.03.

**Figure 2:**
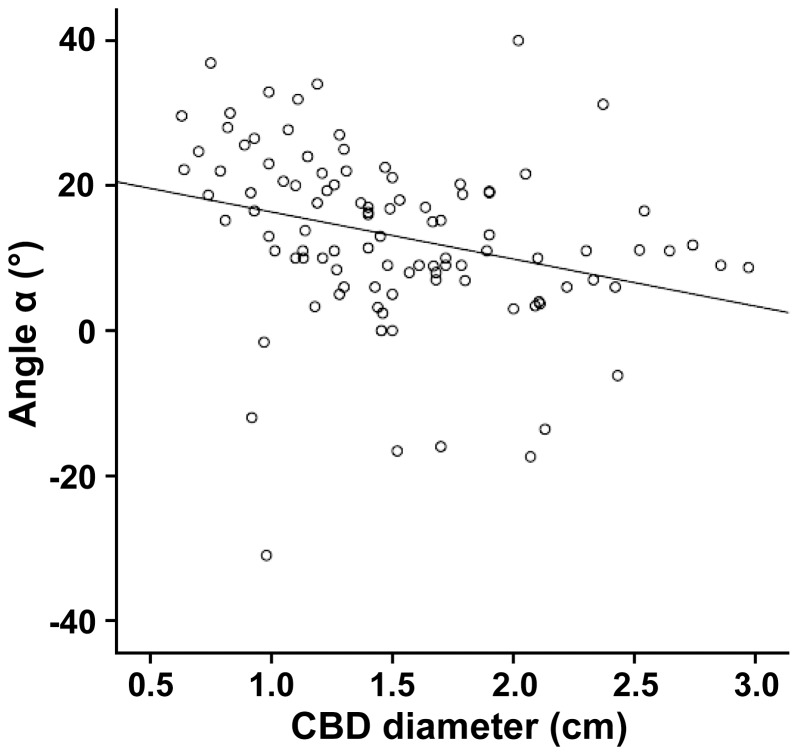
Angle α correlates with the common bile duct diameter. The angle α and the common bile duct (CBD) diameter of all subjects with oblique common bile duct syndrome were plotted on the y and x axes, respectively. Note that both variables display a significant negative correlation (r = −0.29, p = 0.003).

In order to analyse to what extent the bile duct morphology affects the course of biliary disease, OCBD subjects were divided into severe (angle α≤15°) and moderate subgroups (angle α>15°; Table S4). Interestingly, when compared to the moderate subgroup, patients with severe OCBD displayed significantly more dilated CBD (17 vs 13 mm; p<0.0001). This difference was not due to the number of patients who underwent cholecystectomy prior to ERCP, because the rate of cholecystectomies was similar in both subgroups (Table S4). A correlation analysis revealed a negative correlation between the angle α and the CBD diameter (r = −0.29, p = 0.003, Figure 2). No difference was observed in liver enzyme levels, cholestatic parameters or levels of C-reactive protein (Table S4).

We also analysed the impact of OCBDs on the necessity to perform repeated ERCPs. The required ERCPs were divided into “early” and “late”. The early ones occurred within a month after a previous procedure and indicated an incomplete removal of the stones, while the latter ones suggested a “de novo” formation/translocation of concrements into CBD. Of note, OCBD subjects, compared to the control group, required an “early” follow-up ERCP more frequently and the difference was even more apparent in subjects who necessitated at least two “early” follow-up procedures (Table 4). However, no differences in the rates of post-ERCP pancreatitis were noted among the groups. Last but not least, OCBD patients required significantly more frequent “late” ERCPs and the total number of these procedures noted were 38 and 12 in the OCBD and control groups, respectively (Table 4).

**Table 4:** Post-ERCP events.

	“Oblique” CBD	Controls
“Early” ERCP (n), yes/no	45/58^1^	28/75^1^
Multiple “early” ERCP (n), yes/no	14/89^2^	3/100^2^
Post-ERCP pancreatitis (n), yes/no	3/100	4/100
Subjects requiring “late” ERCP (n), yes/no	17/74^3^	8/84^3^
Total “late” ERCPs needed	38	12

“Early” and “late” ERCP was termed an endoscopic retrograde cholangiopancreatography, which occurred within or at least a month after the previous procedure, respectively. Multiple “early” ERCPs refers to the need for at least two “early” ERCPs in one patient. A median follow-up was four years in both groups. CBD: common bile duct.

1 p = 0.01;

2 p = 0.005;

3 p<0.05.

## Discussion

Morphological variants of the biliary tree such as periampullary diverticula or papillary stenoses were shown to predispose to a recurrence of gallstones [5]. To further study the role of the abnormal biliary course in development of gallstones, we identified and further analysed a subgroup of patients presenting with oblique CBD. In our setting, this variant was found in almost 8% of patients. However, these data were obtained in a tertiary care medical centre and further studies are needed to determine the frequency of OCBD in the general population.

Our findings suggest that OCBD may represent an acquired condition. In support of that, OCBD was observed in older patients and was preferentially seen in subjects who previously underwent a cholecystectomy and/or another intervention on the biliary tree. The fact that OCBD patients more often required an open cholecystectomy further supports the hypothesis of an underlying structural bile duct abnormality. However, OCBD does not seem to be a simple consequence of aging, given that no change in the angle α was seen in a subset of patients undergoing consecutive ERCPs. Therefore, further studies are needed to clarify the etiology (i.e., inherited versus acquired) of OCBD and to find out, what additional hits may contribute to development of clinically apparent condition in these subjects.

We also analysed the consequences of OCBD. Our data indicate that it is associated with (i) development of chronic cholestasis and (ii) chronic pancreatitis; (iii) more severe acute cholangitis; (iv) more difficult gallstone removal and with (v) recurrent gallstone formation. The most likely explanation of these findings is that OCBD subjects experience an impaired bile flow, which represents an established risk factor not only for gallstone development but also for the development of chronic pancreatitis [8], [11]. This is further supported by the finding that OCBD subjects had a dilated CBD and the CBD dilation was even more pronounced in patients with severe OCBD. To that end, a dilated CBD represents an established risk factor for gallstone recurrence [9], [12]. However, one has to keep in mind that some of the observations may be in part due to the significant age difference between the control and study group. Future studies are needed to overcome this limitation.

A reduced bile flow might be due to a compromised motility of the biliary tract or due to a biliary obstruction. Given that CBD does not significantly contribute to biliary motility, further studies should analyse whether OCBD associates with gallbladder and/or sphincter of Oddi dysfunction, which represent the major reasons for biliary dysmotility [13]. With respect to biliary obstruction, although patients with biliary stenosis were not included in our analysis, we cannot exclude a presence of transient/functional stenosis, which might have escaped the detection in ERCP. In any case, the horizontal portion of the CBD seems to be of functional relevance, given that gallstones were typically found in this section (not shown).

The observation that OCBD subjects require multiple ERCPs is of particular clinical relevance. First of all, OCBD subjects need more ERCPs to completely remove all stones, a finding which is likely due to a challenging biliary morphology. In addition to that, “late ERCPs” were more frequent in the OCBD subjects and their frequency was significantly above the data reported in the literature [7], [8]. Moreover, several patients required multiple ERCPs during the follow-up. Therefore, a regular surveillance of OCBD patients might be reasonable, especially if they display additional risk factors for stone recurrence such as a dilated bile duct [7]. In this respect, patients with multiple bile stone recurrences were previously suggested to benefit from annual ERCPs [14]. While a regular screening might be helpful, it remains unclear what might be the best treatment option for OCBD patients with recurrent stones. Given the associated biliary dysmorphism, a surgical treatment might be an option. Choledochojejunostomy was already suggested as a treatment option for recurrent common bile duct stones in previous studies [15], [16]. Indeed, five of our OCBD patients with a history of more than five ERCPs underwent a resection of the oblique CBD. A biliodigestive anastomosis with a Roux-en-Y reconstruction and a long loop was performed. These patients were followed up at least one year after surgery and had neither biliary symptoms nor problems due to the surgical treatment. However, one has to keep in mind that the presence of OCBD does not inevitably lead to an incurable disease but merely represents a risk factor for an adverse disease outcome. Only a careful follow-up will tell whether an OCBD patient requires a surgical treatment or whether the OCBD can be managed in a nonsurgical manner.

In summary, OCBD defines a new entity of bile duct abnormality, which is associated with chronic cholestasis, hampers an efficient stone removal and predisposes to recurrence of bile duct stones. Further studies are needed to clarify the pathogenesis of this syndrome and possible treatment strategies. To accurately determine the angle α, these trials should include a magnetic resonance imaging of the bile ducts with a precise three-dimensional reconstruction. Thereby, these studies will enable us to define a cut-off value for angle α which is associated with significant clinical outcomes.

## Supporting Information

Figure S1 **Examples of the radiographs depicting the oblique common bile duct.** Of note, panel (A) depicts a patient in a non-standardised half left position which was used to evaluate the oblique choledochus for further stones. Panel (B) represents the X-ray of the same patient taken in a standardised face-down position, thereby confirming the presence of an oblique bile duct syndrome.
(TIF)

Table S1 **Study cohort.** CBD, common bile duct; SD, standard deviation; ^1^p<0.00001
(DOCX)

Table S2 **Angle α in consecutive endoscopic retrograde cholangiopancreatographies.** Angle α was defined as the minimal angle between the horizontal plane and the CBD. ERCP, endoscopic retrograde cholangiopancreatography.
(DOCX)

Table S3 **Lab values in subjects who underwent cholecystectomy prior to endoscopic retrograde cholangiopancreatography.** Median values are shown. Calculation of statistical significance is based on a two-tailed Mann-U-Whitney test. AP, alkaline phosphatase; GGT, gamma glutamyl transferase; Bili, bilirubin; AST, aspartate transaminase; ALT, alanine transaminase; AP, alkaline phosphatase; d, day; wk, week; mo, month; ERCP, endoscopic retrograde cholangiopancreatography; OB, “oblique” bile duct; Co, Controls. ^1^p = 0.03; ^2^p = 0.006; ^3^p = 0.0001; ^4^p = 0.09; ^5^p = 0.03; ^6^p = 0.01; ^7^p = 0.06; ^8^p = 0.05; ^9^p = 0.02; ^10^p = 0.007; ^11^p = 0.003; ^12^p = 0.02
(DOCX)

Table S4 **Impact of OCBD morphology on cholestatic liver injury during ERCP.** ALT, alanine transaminase; AP, alkaline phosphatase; AST, aspartate transaminase; Bili, bilirubin; CBD, common bile duct; OCBD, “oblique” common bile duct; GGT, gamma glutamyl transferase; SD, standard deviation ^1^p<0.0001
(DOCX)
