# Supplementary figures and images for: Oblique Bile Duct Predisposes to the Recurrence of Bile Duct Stones
Source: PLoS One. 2013 Jan 24;8(1):e54601. doi: 10.1371/journal.pone.0054601 (PMC3554756; doi:10.1371/journal.pone.0054601)

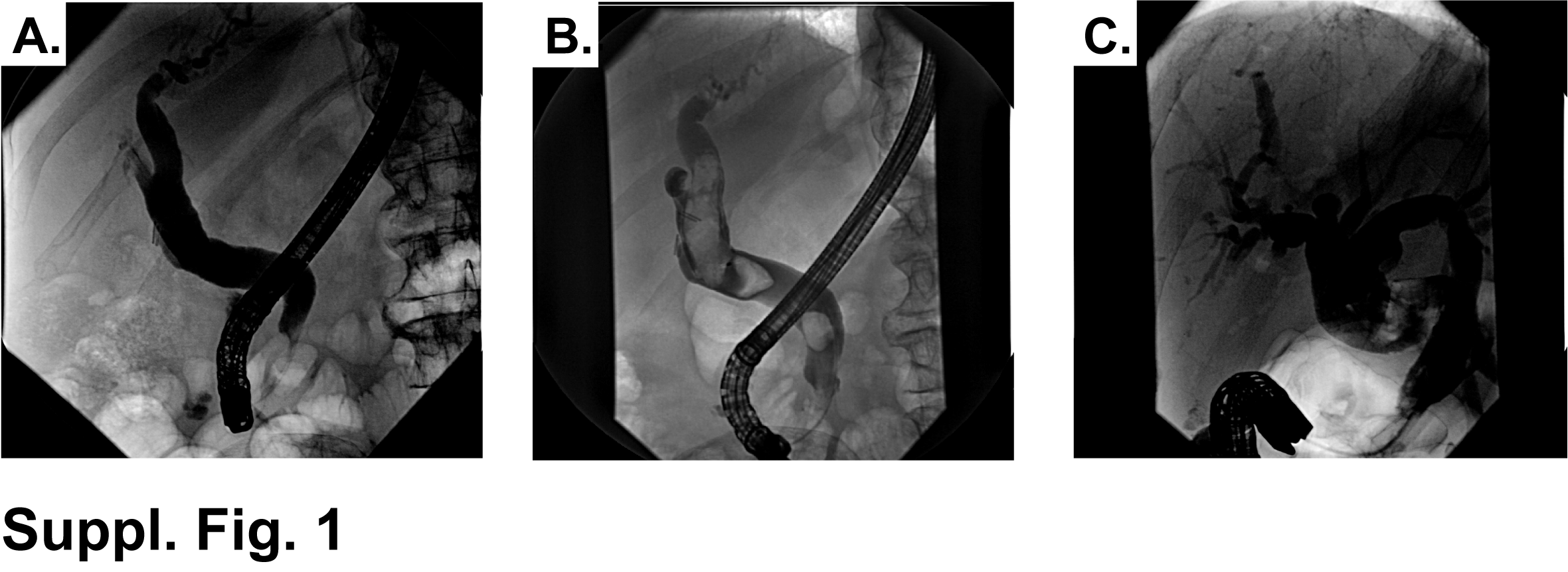

Supplement: Figure S1 — Examples of the radiographs depicting the oblique common bile duct. Of note, panel (A) depicts a patient in a non-standardised half left position which was used to evaluate the oblique choledochus for further stones. Panel (B) represents the X-ray of the same patient taken in a standardised face-down position, thereby confirming the presence of an oblique bile duct syndrome. (TIF) [file pone.0054601.s001.tif]
