# Supplementary material for: Oblique Bile Duct Predisposes to the Recurrence of Bile Duct Stones
Source: PLoS One. 2013 Jan 24;8(1):e54601. doi: 10.1371/journal.pone.0054601 (PMC3554756; doi:10.1371/journal.pone.0054601)
Supplement: Table S1 — Study cohort. CBD, common bile duct; SD, standard deviation; 1p<0.00001 (DOCX) [file pone.0054601.s002.docx]

**Supplementary Table S1. Study cohort**

|  | Analyzed patients | Thereof „oblique“ CBD | Thereof controls |
| --- | --- | --- | --- |
| n | 1307 | 103 | 104 |
| #Male/Female | 555/752 | 40/63 | 35/69 |
| Age±SD | 64,3±17,8 | 71,8±12,5^1^ | 66,8±13,4^1^ |

CBD, common bile duct; SD, standard deviation; ^1^p<0.00001
