# Supplementary material for: Oblique Bile Duct Predisposes to the Recurrence of Bile Duct Stones
Source: PLoS One. 2013 Jan 24;8(1):e54601. doi: 10.1371/journal.pone.0054601 (PMC3554756; doi:10.1371/journal.pone.0054601)
Supplement: Table S2 — Angle α in consecutive endoscopic retrograde cholangiopancreatographies. Angle α was defined as the minimal angle between the horizontal plane and the CBD. ERCP, endoscopic retrograde cholangiopancreatography. (DOCX) [file pone.0054601.s003.docx]

**Supplementary Table S2. Angle α in consecutive endoscopic retrograde cholangiopancreatographies**

| **Patient #** | **Angle α** | | **Time interval (years)** |
| --- | --- | --- | --- |
|  | **1. ERCP** | **2. ERCP** |  |
| 1 | 1 | 7,2 | 1,3 |
| 2 | 22 | 18,8 | 6,6 |
| 3 | -28 | -20 | 7,5 |
| 4 | 13,4 | 7,2 | 2,7 |
| 5 | 5 | -4,8 | 4,0 |
| 6 | 7 | 10 | 3,8 |
| 7 | 19 | 15 | 2,7 |
| 8 | -6,2 | -8 | 4,7 |
| 9 | 6,4 | 5,8 | 7,9 |
| 10 | 2,8 | 3,2 | 3,7 |
| 11 | 19,6 | 16,2 | 7,0 |
| 12 | 20,6 | 21,4 | 1,4 |

Angle α was defined as the minimal angle between the horizontal plane and the CBD.

ERCP, endoscopic retrograde cholangiopancreatography
