# Supplementary material for: Oblique Bile Duct Predisposes to the Recurrence of Bile Duct Stones
Source: PLoS One. 2013 Jan 24;8(1):e54601. doi: 10.1371/journal.pone.0054601 (PMC3554756; doi:10.1371/journal.pone.0054601)
Supplement: Table S3 — Lab values in subjects who underwent cholecystectomy prior to endoscopic retrograde cholangiopancreatography. Median values are shown. Calculation of statistical significance is based on a two-tailed Mann-U-Whitney test. AP, alkaline phosphatase; GGT, gamma glutamyl transferase; Bili, bilirubin; AST, aspartate transaminase; ALT, alanine transaminase; AP, alkaline phosphatase; d, day; wk, week; mo, month; ERCP, endoscopic retrograde cholangiopancreatography; OB, “oblique” bile duct; Co, Controls. 1p = 0.03; 2p = 0.006; 3p = 0.0001; 4p = 0.09; 5p = 0.03; 6p = 0.01; 7p = 0.06; 8p = 0.05; 9p = 0.02; 10p = 0.007; 11p = 0.003; 12p = 0.02 (DOCX) [file pone.0054601.s004.docx]

**Supplementary Table S3. Lab values in subjects who underwent cholecystectomy prior to endoscopic retrograde cholangiopancreatography**

|  | | AP (U/l) | | GGT (U/l) | | Bili (µmol/l) | | AST (U/l) | | ALT (U/l) | | CRP (mg/l) | |
| --- | --- | --- | --- | --- | --- | --- | --- | --- | --- | --- | --- | --- | --- |
|  |  | OB | Co | OB | Co | OB | Co | OB | Co | OB | Co | OB | Co |
| Before  ERCP | >3mo | 92^1^ | 72^1^ | 47^2^ | 20^2^ | 8 | 9 | 29^3^ | 15^3^ | 24 | 17 | 7,4 | 5 |
|  | 1-12wk | 85^4^ | 72^4^ | 35^5^ | 20^5^ | 13 | 9 | 25^6^ | 15^6^ | 16 | 17 | 18 | 5 |
|  | 4-7d | 150 | 97 | 194 | 142 | 11 | 14 | 34 | 26 | 34 | 36 | 10 | 9 |
|  | 1-2d | 179 | 172 | 307 | 327 | 29 | 23 | 34 | 104 | 64^7^ | 153^7^ | 14 | 12 |
| ERCP | | 141 | 154 | 186 | 336 | 44 | 31 | 53^8^ | 136^8^ | 89^9^ | 243^9^ | 75 | 52 |
| After ERCP | 1d | 149 | 155 | 288 | 362 | 31 | 24 | 76 | 76 | 104 | 159 | 57 | 122 |
|  | 2d | 146 | 143 | 332 | 241 | 34 | 21 | 26 | 65 | 64 | 137 | 88 | 59 |
|  | 3d | 155 | 215 | 177 | 276 | 21 | 24 | 19^10^ | 56^10^ | 42^11^ | 140^11^ | 21 | 34 |
|  | 4-7d | 227 | 173 | 192 | 225 | 15 | 12 | 29 | 43 | 34^12^ | 104^12^ | 34 | 28 |
|  | 1-12wk | 101 | 88 | 83 | 53 | 9 | 10 | 22 | 25 | 21 | 34 | 5 | 7 |
|  | >3mo | 92 | 74 | 47 | 33 | 8 | 8 | 29 | 26 | 24 | 31 | 10 | 7 |

Median values are shown. Calculation of statistical significance is based on a two-tailed Mann-U-Whitney test.

AP, alkaline phosphatase; GGT, [gamma glutamyl transferase](http://www.thefreedictionary.com/gamma+glutamyl+transferase+%28GGT%29); Bili, bilirubin; AST, aspartate transaminase; ALT, alanine transaminase; AP, alkaline phosphatase; d, day; wk, week; mo, month; ERCP, endoscopic retrograde cholangiopancreatography; OB, “oblique” bile duct; Co, Controls

^1^p=0.03; ^2^p=0.006; ^3^p=0.0001; ^4^p=0.09; ^5^p=0.03; ^6^p=0.01; ^7^p=0.06; ^8^p=0.05; ^9^p=0.02; ^10^p=0.007; ^11^p=0.003; ^12^p=0.02
