# Supplementary material for: Oblique Bile Duct Predisposes to the Recurrence of Bile Duct Stones
Source: PLoS One. 2013 Jan 24;8(1):e54601. doi: 10.1371/journal.pone.0054601 (PMC3554756; doi:10.1371/journal.pone.0054601)
Supplement: Table S4 — Impact of OCBD morphology on cholestatic liver injury during ERCP. ALT, alanine transaminase; AP, alkaline phosphatase; AST, aspartate transaminase; Bili, bilirubin; CBD, common bile duct; OCBD, “oblique” common bile duct; GGT, gamma glutamyl transferase; SD, standard deviation 1p<0.0001 (DOCX) [file pone.0054601.s005.docx]

**Supplementary Table S4. Impact of OCBD morphology on cholestatic liver injury during ERCP**

|  | Moderate OCBD (n=46) | Severe OCBD (n=56) |
| --- | --- | --- |
| CBD diameter (mm) mean±SD  Prior cholecystectomy (n), yes/no  Bili (µM/ml) mean±SD  AST (U/l) mean±SD  ALT (U/l) mean±SD  GGT (U/l) mean±SD  AP (U/l) mean±SD  CRP (mg/l) mean±SD | 12,8±4,5^1^  30/18  50±60  115±162  112±143  406±622  228±308  66±83 | 17,1±5,2^1^  22/19  45±48  144±210  167±206  392±557  236±171  55±71 |

ALT, alanine transaminase; AP, alkaline phosphatase; AST, aspartate transaminase; Bili, bilirubin; CBD, common bile duct; OCBD, “oblique” common bile duct; GGT, [gamma glutamyl transferase](http://www.thefreedictionary.com/gamma+glutamyl+transferase+%28GGT%29); SD, standard deviation

^1^p<0.0001
